# Klatskin tumor diagnosed concurrently with IgG4 related sclerosing cholangitis: A case report

**DOI:** 10.1097/MD.0000000000021936

**Published:** 2020-08-21

**Authors:** Ha Won Hwang, Jin-Seok Park, Seok Jeong, Don Haeng Lee, Suk Jin Choi

**Affiliations:** a Digestive Disease Center, Department of Internal Medicine; b Department of pathology, Inha University School of Medicine, Incheon, Republic of Korea.

**Keywords:** cholangiocarcinoma, IgG4-related disease, IgG4-related sclerosing cholangitis

## Abstract

**Rationale:**

IgG4-related disease (IgG4-RD) is a systemic disease that can involve various organs and is characterized by the infiltrations of IgG4-positive plasma cells and lymphocytes, fibrosis, and elevated serum IgG4 levels. IgG4-related sclerosing cholangitis (IgG4-RSC) is a subtype of IgG4-RD. No certain relationship between IgG4-RSC and cholangiocarcinoma has been established as yet, and there have been few reports of the simultaneous diagnosis of IgG4-RSC and cholangiocarcinoma.

**Patient concerns:**

A 76-year-old male visited our gastroenterology department due to the recent occurrence of pruritus and jaundice.

**Diagnosis:**

Computed tomography (CT) scan showed ductal wall swelling and enhancement from both intrahepatic duct confluence to the common bile duct, upper biliary dilatation, and accompanying autoimmune pancreatitis (a sub type of IgG4-RD). Biopsy of the distal common bile duct by endoscopic retrograde cholangiopancreatography (ERCP) resulted in a diagnosis of IgG4-RSC. Subsequently, adenocarcinoma was identified by repeated cytology of bile juice. Finally, Klatskin tumor type IIIA and IgG4-RSC were concurrently diagnosed.

**Interventions:**

IgG4-RSC was treated with steroid and Klatskin tumors by gemcitabine + cisplatin chemotherapy.

**Outcomes:**

The jaundice had improved and CT showed substantial improvement of the intrahepatic duct dilatation.

**Lessons:**

IgG4-RSC and cholangiocarcinoma are easily confused, but their treatments are quite different, and thus, care must be taken during diagnosis. Furthermore, these 2 diseases may co-exist. Therefore, even if IgG4-RSC is diagnosed first, the possibility of accompanying cholangiocarcinoma should be thoroughly investigated.

## 1 Introduction

IgG4-related disease (IgG4-RD) is characterized by the infiltrations of IgG4-positive plasma cells and lymphocytes, fibrosis, and an elevated serum IgG4 level. IgG4-RD is a relatively recently recognized disease, and the mechanism responsible for its pathogenesis has not been established.^[1]^ Many diseases previously considered to be limited to one organ are now considered to belong to the IgG4-RD spectrum, and involvements of various organs including the pancreatobiliary system, salivary glands, kidneys, and lymph nodes have been reported.^[2]^ In the majority of cases, IgG4-RDs respond well to steroids, although immunosuppressants can be used when response is inadequate or steroids are unavailable.

IgG4-related sclerosing cholangitis (IgG4-RSC) occurs when IgG4-RD invades the biliary tract and can be confused with other benign or malignant diseases such as primary sclerosing cholangitis (PSC) or cholangiocarcinoma. However, their treatments are quite different, and thus, care must be taken during diagnosis. In fact, many case reports have mentioned cases in which IgG4-RSC and cholangiocarcinoma were confused.^[3,4]^ Interestingly, few cases of IgG4-RSC and cholangiocarcinoma co-existence have been reported.^[5–7]^ The relationship between IgG4-RD and the malignancy is not yet clear. Although several studies suggest that IgG4-RD may be a risk factor for malignancy,^[8–11]^ the mechanism has not been revealed. Here, we report such a case of coexistent IgG4-RSC and Klatskin tumor in a 76-year-old male and discuss the relationship between the 2 diseases.

## 2 Case presentation

Patient has provided informed consent for publication of the case. A 76-year-old male visited our gastroenterology department due to a recent deterioration in systemic pruritus and jaundice. The patient had diabetes mellitus, had been treated for autoimmune pancreatitis (a subtype of IgG4-RD) 3 years previously, and had undergone cholecystectomy and stent insertion 2 years previously due to cholecystitis and intrahepatic duct stricture. The patient had an acute ill-looking appearance and systemic jaundice. Vital signs were as follows; blood pressure 140/80, pulse rate 73/minute, respiratory rate 20/minute, and body temperature 36.6°C.

**Figure 1:**
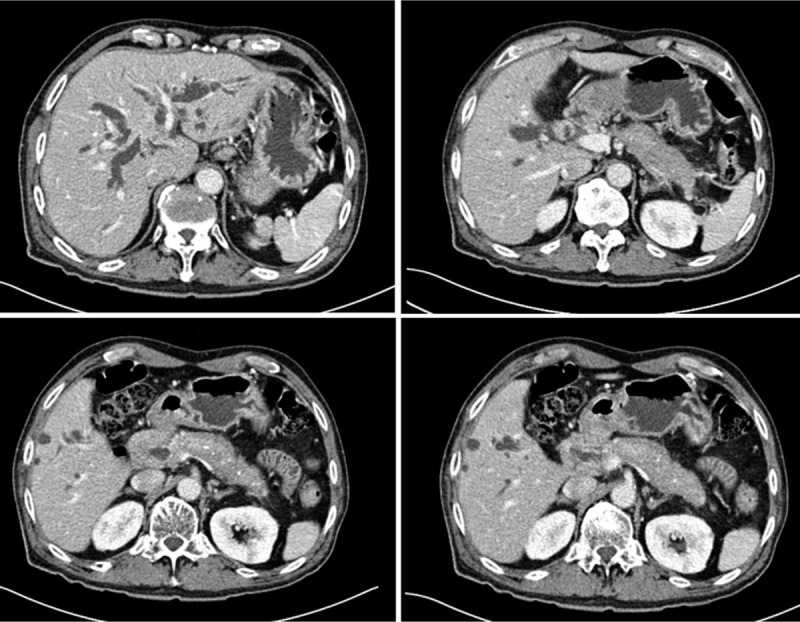
Computed tomography showed ductal wall swelling and enhancement from both intrahepatic duct confluence to the common bile duct, upper biliary dilatation. In addition, the pancreas was swollen and showed low attenuation at its periphery.

**Figure 2:**
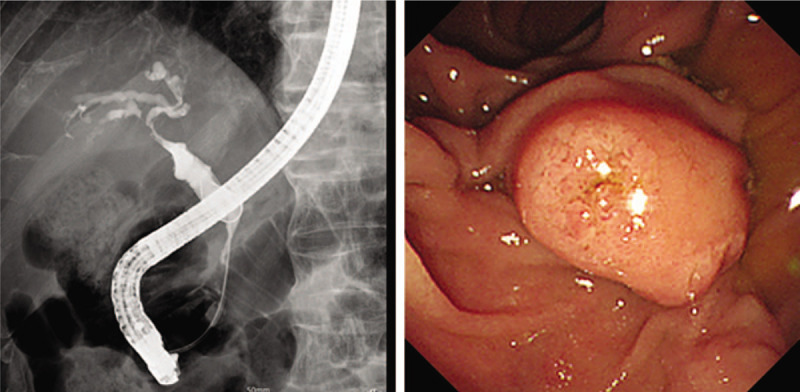
Endoscopic retrograde cholangiopancreatography showed obstruction of the common hepatic duct. The distal common bile duct was also presumed to be obstructed.

**Figure 3:**
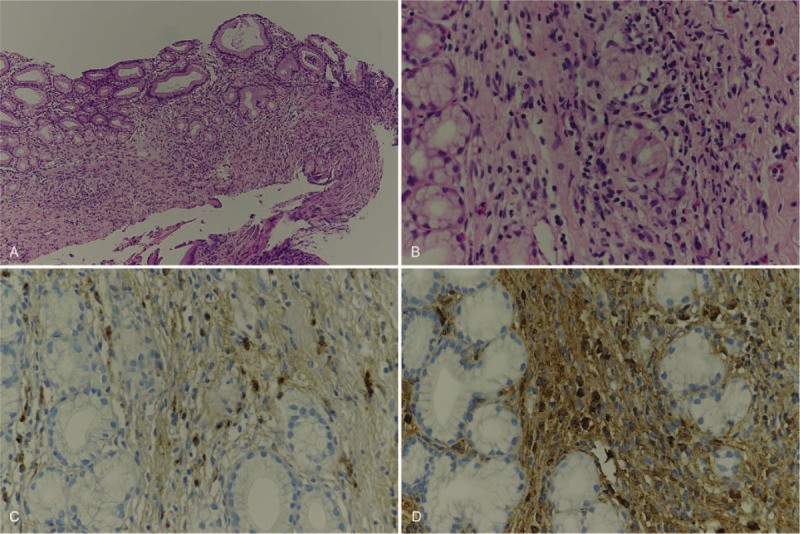
(A) Many inflammatory cells and fibrosis were present in the lamina propria (original magnification ×100). (B) Inflammatory cells included many plasma cells and lymphocytes (original magnification ×400). (C, D) Immunohistochemical staining for IgG and IgG4; many plasma cells were positive for IgG and IgG4. The IgG4/IgG ratio was >0.4 and the number of IgG4 positive cells/high-power field exceeded 10 (original magnification ×400).

**Figure 4:**
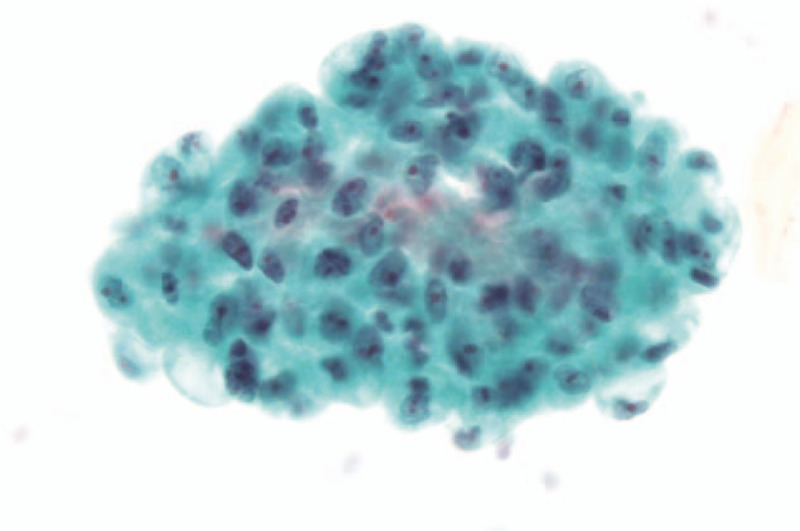
Brushing cytology test revealed clusters of atypical cells with large vesicular nuclei and pale open chromatin, nuclear membrane irregularity, variable nuclear sizes, and cellular polarity, which prompted a diagnosis of adenocarcinoma.

The patient was admitted and laboratory tests showed; total bilirubin was increased to 9.5 mg/dL, direct bilirubin to 8.0 mg/dL, aspartate aminotransferase to 74 IU/L, alanine aminotransferase to 136 IU/L, alkaline phosphatase to 506 IU/L, gamma glutamyl transpeptidase to 605 IU/L, and C-reactive protein to 0.72 mg/dL. Amylase and lipase were normal, but CA19–9 (a tumor marker) was high at 328 U/mL. Computed tomography (CT) revealed ductal wall swelling and enhancement from both intrahepatic duct confluence to the common bile duct, and upper biliary dilatation. In addition, the pancreas was swollen and displayed low attenuation in its periphery (Fig. 1). Endoscopic retrograde cholangiopancreatography (ERCP) showed obstruction of the common hepatic duct and presumed obstruction of the distal common bile duct (Fig. 2). We performed endoscopic sphincterotomy. A brushing cytology test on the common hepatic duct and biopsy of the distal common bile duct were conducted. Subsquently, endoscopic retrograde biliary drainage and endoscopic nasobiliary drainage were performed. Biopsy revealed chronic nonspecific inflammation with diffuse positivity for IgG4 (Fig. 3). No malignancy was observed in the biopsy, but adenocarcinoma was confirmed by brushing cytology testing of the common hepatic duct (Fig. 4). In addition, IgG-related blood tests showed high levels of IgG 2563 mg/dL and IgG subclass IV 782 mg/dL. As a result, the patient was diagnosed to have concurrent Klatskin tumor type IIIA and IgG4-RSC.

Prednisolone was started at 0.6 mg/kg/day to address the IgG4-RSC, and about a week later, laboratory tests showed; total bilirubin had fallen to 3.9 mg/dL, direct bilirubin to 3.6 mg/dL, aspartate aminotransferase to 42 IU/L, alanine aminotransferase to 60 IU/L, and alkaline phosphatase to 235 IU/L. Prednisolone was tapered as the patient improved. Klatskin tumors were treated with gemcitabine + cisplatin chemotherapy. After chemotherapy, the intrahepatic duct dilatation was obviously improved and the common bile duct appeared normal by follow-up CT. Subsequently, the patient was followed up for about 2 years through outpatient clinic. There was no exacerbation of the diseases during the follow-up period.

## 3 Discussion

IgG4-RSC can be easily confused with PSC or cholangiocarcinoma, and thus, it is difficult to distinguish between these diseases. However, the treatment and prognosis of these diseases are very different, and thus, it is important that a differential diagnosis be made. Many case reports have mentioned cases in which IgG4-RSC and cholangiocarcinoma were confused.^[3,4]^ These case reports demonstrate the difficulty in distinguishing between IgG4-RSC and cholangiocarcinoma. Elevated serum IgG and IgG4 levels, IgG4-related involvement of other organs (eg, autoimmune pancreatitis), lower bile duct stenosis, and bile duct wall thickening in areas without stenosis strongly suggest IgG4-RSC. However, obstructive jaundice, elevated serum CA 19–9, and obstruction of the hilar bile duct by ERCP suggest cholangiocarcinoma. These features can help to differentiate the 2 diseases,^[12]^ but they do not always apply and there are cases where both diseases coexist. Therefore, it is critical that clinical findings, blood tests, imaging tests, and biopsies be judiciously assessed, and if necessary, repeat biopsy be performed.

In the described case, IgG4-RSC and cholangiocarcinoma were not confused but diagnosed simultaneously, and such cases have rarely been reported.^[5–7]^ Since IgG4-RSC and cholangiocarcinoma may coexist, a final diagnosis should not be made prematurely even if IgG4-RSC is suspected and repeat biopsies should be undertaken to exclude the possibility of accompanying cholangiocarcinoma. In our patient, no malignancy was found by the first biopsy and brushing cytology test. However, to exclude cholangiocarcinoma, an additional biopsy and brushing cytology test was performed by ERCP. Finally, brushing cytology testing of the common hepatic duct proved to be positive for adenocarcinoma.

The relationship between IgG4-RSC and cholangiocarcinoma has not been clearified. But, several recent studies have suggested that IgG4-RSC may be a risk factor for cholangiocarcinoma. Much research has addressed the possibility that IgG4-RDs, such as IgG4-RSC, are a risk factor for malignancy.^[10,11]^ In a study by Yamamoto et al, in which 106 patients diagnosed with IgG4-RD were analyzed, malignancies were observed in 11 patients (10.4%), either at diagnosis or during a follow-up period of 3 years. The standardized incidence ratio for these malignancies in IgG4-RD was 383.0, which was higher than that for the general population.^[10]^ In a prospective UK cohort study conducted by Huggett et al, in which 115 patients with autoimmune pancreatitis or IgG4-RSC were followed for an average of 33 months, malignancy occurred in 11% shortly before or after autoimmune pancreatitis or IgG4-RSC was diagnosed. The risk of any cancer at diagnosis or during follow-up when compared with matched national statistics was increased (odds ratio = 2.25).^[11]^ Indeed, cases in which IgG4-RSC may have acted as a risk factor of cholangiocarcinoma development have been reported.^[9]^ Similarly, cases where autoimmune pancreatitis may have acted as a risk factor of malignancy development have also been reported.^[8]^

It is not clear whether IgG4-RSC is a risk factor for cholangiocarcinoma, but there are 2 main reasons why it might be. First, sustained inflammatory conditions associated with IgG4-RD are considered to create immunological environments favorable for cancer development.^[13]^ Second, the immunological environments associated with IgG4-RD can interfere with immune responses that inhibit cancer development. The immunologic characteristic of IgG4-RD is the activation of type 2 helper T (Th2) cells and regulatory T (Treg) cells.^[14]^ Autoimmunity or infection may act as an immunologic trigger in IgG4-RD. Interleukins 4, 5, 10, and 13 and transforming growth factor β (TGF-β) are overexpressed through an immune reaction in which Th2 cells predominate, followed by activation of Treg cells. As a result, serum IgG4 increases and IgG4-positive plasma cells and lymphocytes infiltrate and damage organs. Furthermore, TGF-β may play an important role in the promotion of fibrosis.^[1]^ Treg cells which are activated in IgG4-RD suppress immune responses and interleukin-10 is a regulatory cytokine that broadly functions as an immune inhibitory cytokine to support tumor growth. This suggests that Treg cells play a role in the progression and metastasis of various malignant tumors.^[13]^ In a study of pancreatic ductal adenocarcinoma by Hiraoka et al, the prevalence of Treg cells in CD4+ T cells was significantly increased in the ductal adenocarcinomas compared with that in the stroma of nonneoplastic inflammation. And the prevalence of Treg cells in CD4+ T cells increased significantly during the progression of premalignant lesions. Furthermore, a better prognosis was observed in patients with a low prevalence of Treg cells in CD4+ T cells. These findings suggest that Treg cells play a role in controlling the immune response against pancreatic ductal carcinoma from the premalignant stage to established cancer.^[15]^ Based on these findings, it is thought that IgG4-RSC may promote the development of cholangiocarcinoma.

In the described case, it was difficult to evaluate the causal relationship between IgG4-RSC and cholangiocarcinoma because they were diagnosed at the same time. However, considerations of a medical history of diagnosed autoimmune pancreatitis three years previously suggest the progress of IgG4-RSC may have sustained that long, and sustained IgG4-RSC may have been responsible for the development of cholangiocarcinoma. Given that IgG4-RSC may be a risk factor for cholangiocarcinoma, it is important that IgG4-RSC be diagnosed early and treated appropriately in the prevention of cholangiocarcinoma.

## 4 Conclusion

We report a case of concurrent IgG4-RSC and cholangiocarcinoma, which were diagnosed simultaneously. IgG4-RSC is a subtype of IgG4-RD and can be confused with PSC and cholangiocarcinoma, but its treatment and prognosis are quite different, and thus differential diagnosis is important. In addition, many studies have shown that IgG4-RSC may be a risk factor for cholangiocarcinoma, and since IgG4-RSC and cholangiocarcinoma may coexist, even if IgG4-RSC is diagnosed first, it is important that repeated tests be conducted to ensure that it is not accompanied by cholangiocarcinoma. IgG4-RD was only recognized as a disease entity relatively recently and its mechanism is unclear. Accordingly, we suggest that the mechanism responsible for the development of IgG4-RD and the relationship between this disease and malignancy be further researched.

## Author contributions

**Conceptualization:** Ha Won Hwang, Jin-Seok Park.

**Investigation:** Ha Won Hwang.

**Resources:** Seok Jeong, Don Haeng Lee, Suk Jin Choi.

**Supervision:** Jin-Seok Park.

**Visualization:** Suk Jin Choi.

**Writing – original draft:** Ha Won Hwang.

**Writing – review & editing:** Jin-Seok Park, Seok Jeong.

## References

[1] Stone JH Zen Y Deshpande V IgG4-related disease. N Engl J Med 2012;366:539–51. 22316447 10.1056/NEJMra1104650

[2] Saeki T Saito A Hiura T Lymphoplasmacytic infiltration of multiple organs with immunoreactivity for IgG4: IgG4 related systemic disease. Internal Medicine 2006;45:163–7. 16508232 10.2169/internalmedicine.45.1431

[3] Azeem N Ajmera V Hameed B Hilar cholangiocarcinoma associated with immunoglobulin G4-positive plasma cells and elevated serum immunoglobulin G4 levels. Hepatol Commun 2018;2:349–53. 29619414 10.1002/hep4.1164 PMC5880190

[4] Nguyen-tat M Gamstatter T Marquardt JU IgG4-related sclerosing cholangitis mimicking cholangiocarcinoma. Z Gastroenterol 2012;50:1008–12. 22965631 10.1055/s-0031-1299451

[5] Hyoung-Chul O Jae Gyu K Jeong Wook K Early bile duct cancer in a background of sclerosing cholangitis and autoimmune pancreatitis. Intern Med 2008;47:2025–8. 19043254 10.2169/internalmedicine.47.1347

[6] Zhang YA Shen XZ Zhu JM Extensive metastatic cholangiocarcinoma associated with IgG4-related sclerosing cholangitis misdiagnosed as isolated IgG4-related sclerosing cholangitis: a case report and literature review. Medicine (Baltimore) 2015;94:e2052. 26559312 10.1097/MD.0000000000002052 PMC4912306

[7] Straub BK Esposito I Gotthardt D IgG4-associated cholangitis with cholangiocarcinoma. Virchows Arch 2011;458:761–5. 21484428 10.1007/s00428-011-1073-2

[8] Loos M Esposito I Hedderich DM Autoimmune pancreatitis complicated by carcinoma of the pancreatobiliary system: a case report and review of the literature. Pancreas 2011;40:151–4. 21160372 10.1097/MPA.0b013e3181f74a13

[9] Koopman KE Bloemena E Kazemier G Immunoglobulin G4-mediated sclerosing cholangitis as a risk factor for cholangiocarcinoma: a case report. Mol Clin Oncol 2016;5:786–8. 28105357 10.3892/mco.2016.1040 PMC5228479

[10] Yamamoto M Takahashi H Tabeya T Risk of malignancies in IgG4-related disease. Mod Rheumatol 2012;22:414–8. 21894525 10.1007/s10165-011-0520-x

[11] Huggett MT Culver EL Kumar M Type 1 autoimmune pancreatitis and IgG4-related sclerosing cholangitis is associated with extrapancreatic organ failure, malignancy, and mortality in a prospective UK cohort. Am J Gastroenterol 2014;109:1675–83. 25155229 10.1038/ajg.2014.223 PMC4552254

[12] Tabata T Kamisawa T Hara S Differentiating immunoglobulin g4-related sclerosing cholangitis from hilar cholangiocarcinoma. Gut Liver 2013;7:234–8. 23560161 10.5009/gnl.2013.7.2.234 PMC3607779

[13] Harada K Nakanuma Y Cholangiocarcinoma with respect to IgG4 Reaction. Int J Hepatol 2014;2014:803876. 25132998 10.1155/2014/803876 PMC4123618

[14] Zen Y Fujii T Harada K Th2 and regulatory immune reactions are increased in immunoglobin G4-related sclerosing pancreatitis and cholangitis. Hepatology 2007;45:1538–46. 17518371 10.1002/hep.21697

[15] Hiraoka N Onozato K Kosuge T Prevalence of FOXP3+ regulatory T cells increases during the progression of pancreatic ductal adenocarcinoma and its premalignant lesions. Clin Cancer Res 2006;12:5423–34. 17000676 10.1158/1078-0432.CCR-06-0369

